# HPV testing on vaginal/cervical nurse-assisted self-samples versus clinician-taken specimens and the HPV prevalence, in Adama Town, Ethiopia

**DOI:** 10.1097/MD.0000000000016970

**Published:** 2019-08-30

**Authors:** Eshetu Lemma Haile, Simoens Cindy, Benoy Ina, Gurja Belay, Van geertruyden Jean-Pierre, Ransom Sharon, Lebelo Ramokone Lisbeth, Bogers Johannes Paul

**Affiliations:** a University of Antwerp, Antwerpen, Belgium; b Addis Ababa University, Addis Ababa, Ethiopia; c Algemeen Medisch Laboratorium (AML), Sonic Healthcare, Antwerpen, Belgium; d International Partnership for Reproductive Health, Bryan, OH; e Sefako Makgatho Health Sciences University (SMU), Gauteng Province, South Africa.

**Keywords:** cervical cancer, clinician-taken, ethiopia, HPV, liquid cytology, nurse, qPCR, self-sampling, thinPrep preservCyt solution

## Abstract

This study aimed to determine the feasibility of vaginal/cervical nurse-assisted self-sampling (NASS) and the agreement between human papilloma virus (HPV) tests on self-samples versus clinician-taken (CT) specimens.

Women participated voluntarily for cervical cancer screening at St. Aklesia Memorial Hospital. Eighty-three women provided a total of 166 coupled self-taken and CT specimens collected. Specimens were stored at room temperature for a maximum of 10 months and analyzed using validated the RIATOL qPCR HPV genotyping test, a quantitative polymerase chain reaction (qPCR) high-throughput HPV E6, E7 assay. The average age of the participating women was 32 years. Seventy-three women (87.9%) felt that NASS was easy to use. An overall HPV, high-risk (HR) HPV, and low-risk HPV prevalence was 22.7% (15/66), 18.2% (12/66), and 6.1% (4/66), respectively. The overall HR HPV prevalence was 17.2% (NASS) and 15.5% (CT). The most prevalent HPV type was HPV51; HPV 16 was only detected in 1 woman (CT+NASS) and HPV18 only in 1 woman (CT). The overall measurement agreement between self-taken and CT samples was moderate with a kappa value of 0.576 (*P* < .001). Lifetime partnered with >2 men were associated with HR HPV positivity (*P* < .001). There was a strong statistical association between HR HPV positivity and visual inspection with acetic acid- positive (*P* < .001). The NASS for HPV testing could be seen as an alternative option and might be acceptable to Ethiopian women. The overall HR HPV prevalence was comparable with Sub-Saharan countries in the general population.

## 1 Introduction

Invasive cervical cancer (ICC) is the fourth most frequent malignancy and cause of death in women suffering from cancer worldwide.^[1]^ In Ethiopia, ICC is even at the second place among women between 15 and 44 years of age. Ethiopia has 31.5 million women aged 15 years and older and 7.095 women were diagnosed yearly with ICC of whom 4.732 died from the disease (estimates for 2012). Currently, there are only sparse data on the human papilloma virus (HPV) burden in the general population of Ethiopia.^[2]^

A study in Nigeria, for example, found 93% participation in the self-sampling (SS) arm, compared to only 56% in the hospital-collection arm.^[3]^ Another study in Sub-Saharan Africa indicated a comparable HPV prevalence for self- (14.6%) and physician (12.7%) samples, so similar accuracy of the test on both sampling methods.^[4]^ A study in Madagascar showed absolute acceptance (100%) of SS (with a flocked swab) followed by HPV testing as cervical cancer screening method.^[5]^

Available data indicate that the HPV prevalence in Ethiopia among women with normal cervical cytology varies between 15.9% and 17.5%, and 96.6% of the ICCs are attributed to HPV16 (78.4%) and 18 (18.2%).^[2]^

Cervical cancer develops over a long period of time through precursor lesions. These lesions can be detected by (cytological or visual) screening, and progression toward cancer can then be stopped by treatment (ablation or excision) in an early phase.^[6]^ Currently in Ethiopia, 200 health facilities are providing visual inspection with acetic acid (VIA) screening followed by cryotherapy (ablative treatment technique),and >52,000 women were screened in 2016/17. Of the 20 million women eligible for screening, only 0.3% of them screened. In addition, Loop electrosurgical excision procedure service was scaled up from 5 to 15 hospitals and the Federal Ministry of Health (FMoH) is working to expand VIA screening and cryotherapy into 823 districts.^[7]^ However, more efforts or other screening techniques are urgently necessary to scale up the cervical cancer screening coverage in Ethiopia.

This study aimed to determine the feasibility and acceptability of vaginal/cervical nurse-assisted self-sampling (NASS) and the agreement between HPV test on self-samples versus clinician-taken (CT) specimens in the Ethiopian population.

## 2 Methodology

The study aimed to determine the feasibility of vaginal/cervical NASS and the agreement between HPV testing on self-samples versus CT specimens.

The study was conducted in Adama Town, Oromia region, having a total population of 1,356,342 people of whom 659,992 are females. The St. Aklesia Memorial Hospital (SAMH), located in Adama Town, is a private hospital with a long-time history and expertise in cervical cancer screening.

To reach in an efficient way to a lot of women for recruitment in the study, radio calls and face–to-face interactions were organized. Through these channels, women were encouraged to schedule an appointment for cervical cancer screening approximately 2 weeks (10–18 days) after the first day of their last menstrual period. Also, women visiting the hospital for reproductive health-related issues were called for participation in the study.

Women were eligible if they were 20 years or older, had an intact uterus, had no history of cervical cancer, were mentally competent, and able and willing to provide informed consent. Based on the upset of this study, a cross-sectional and probability sampling technique was used. The minimum calculated sample size of this study was 73.

Women who were interested in participating in the study were given following instructions: no douche 48 hours before the test; no use of tampons, birth control foams, jellies, or other vaginal creams or vaginal medications for 48 hours before the test and also advised to refrain from intercourse 48 hours before the test.

After signing an informed consent document, women were subjected to 2 ways of sample collection, both performed within the clinic: NASS with supervision; CT specimens, that is, a physician collected the samples according to the standard procedure of the clinic. Women were also asked to fill in a questionnaire.

### 2.1 NASS at the clinic

Women were invited to the private area of the clinic and were given verbal and printed diagrammatic instructions by the trained nurse for collecting the vaginal specimen. When the women confirmed that all instructions were clear, the nurse opened the collection kit and handed over the collection devices (in sequence order of spatula followed by cytobrush) to the woman

The vaginal fornix and ectocervix were sampled before the endocervix. To start the NASS, women were instructed to take a sample of the ectocervix using a plastic spatula, without speculum. The women were asked to insert the spatula, laying on the bed, into their vagina and to rotate 3 times at 360 degree, to remove and to handover the collection device to the nurse. The nurse then rinsed the spatula into a labeled vial with ThinPrep PreservCyt solution.

In the next step, the nurse provided the cytobrush to the woman to sample the endocervix. It was inserted by the woman herself until it met with resistance, rotated 45 to 90 degree, removed, and handed over to the nurse. The nurse inserted the cytobrush sample into the same ThinPrep PreservCyt labeled vial. This procedure was not involving any invasive steps rather noninvasive simple and easy collection techniques. Collected samples were kept at 22°C (room temperature) for about 10 months, until shipment and processing.

### 2.2 CT sample at the clinic

The clinicians collected cervical samples according to standard protocols, that is, both ectocervix and endocervix samples were collected with a cytobrush and rinsed in a labeled vial with ThinPrep PreservCyt solution. Collected samples were kept at 22°C (room temperature) for about 10 months, until shipment and processing.

### 2.3 VIA

After the NASS and the CT sample, all women underwent VIA. A woman was classified as VIA-positive when acetowhite lesions were visualized by the clinician. All VIA-positive women were eligible for cryotherapy and were treated.

### 2.4 Ethical clearance

The ethical committee of the College of Natural Sciences, Addis Ababa University, has examined the project and approved it. The SAMH Hospital also approved the project. All women signed informed consent before enrolment in the study.

### 2.5 Laboratory

Both CT and NASS specimens were tested for presence of HPV with the RIOTOL quantitative polymerase chain reaction (qPCR) HPV genotyping test (Algemeen Medisch Laboratorium, Belgium). This method is validated and ISO-certified laboratory-developed (LDT) high-throughput HPV test detects 14 high-risk (HR) HPV types, that is, 16, 18, 31, 33, 35, 39, 45, 51, 52, 56, 57, 58, 59, 66, and 68, four intermediate/low-risk HPV (LR HPV) types, that is, 6, 11, 53, and 67; 68 and a cell control.^[8,9]^ Samples <10 cell/μL are considered as invalid and reported as samples of poor quality.

### 2.6 Data source and analysis

Quantitative data were collected and for some of the demographic variables were decoded accordingly. Any missed variable identified during the collection of data, the supervisor was responsible to follow-up the patients and correct it accordingly. Statistical analysis was performed using the Statistical Package for the Social Sciences (SPSS) version 20 software. The overall measurement agreement between self- and clinician-collected samples was calculated with a kappa value. The dependent variable was HPV outcome and independent variables are sociodemographic. Pearson *χ*^2^ test and 95% confidence interval (CI) were used and statistically significant if the *P* value was ≤0.05.

## 3 Results

A total of 83 eligible women were enrolled between October 2015 and July 2016 at SAMH hospital, Adama, Oromia region, Ethiopia. The study had no missed data or variables.

### 3.1 Patient demographics

The average age of eligible women was 32 years and the youngest was 20 and the oldest 65 years. Forty-seven women (56.6%) had an education level below grade 10 (high school); 33.7% (28/83) and 27.7% (23/83) of the study population were laborers and housewives, respectively. Seventy-one women (85.5%) were married at the time of the study and 69.9% had 1 lifetime partner. A total of 80.7% (67/83) had gravidity ≥1 and 7.5% (5/67) of these women had a spontaneous abortion before. Women who used birth control and smoking cigarettes were 39.8% (33/83) and 18.1% (15/83), respectively. Four (4.8%) women reported being infected with HIV at the time of the study (Table 1).

**Table 1:**
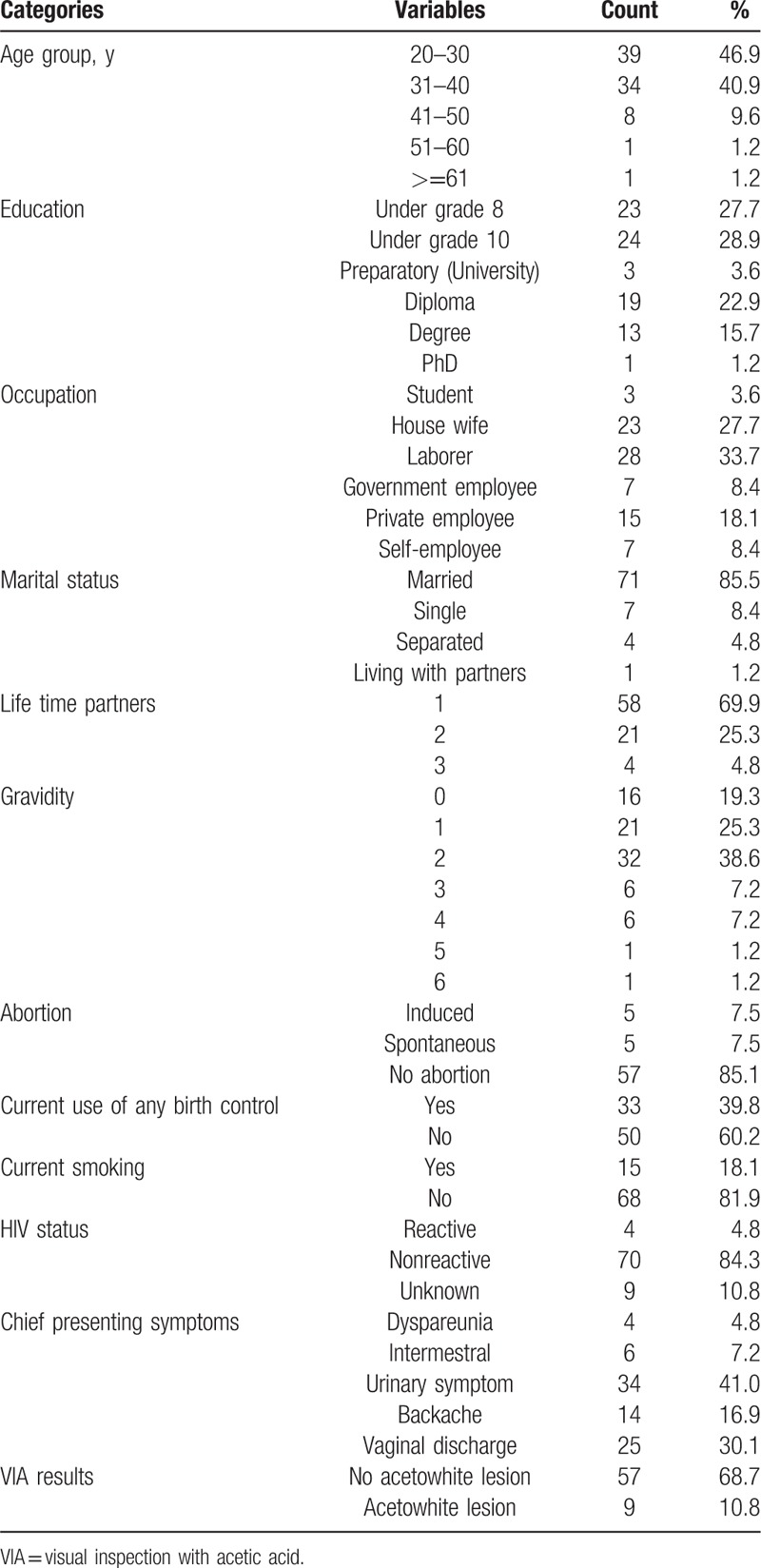
Characteristics of women enrolled in our cervical cancer screening study (N = 83), between October 2015 and July 2016, at SAMH hospital, Adama, Oromia region, Ethiopia.

### 3.2 Acceptance and feasibility of self-samples

Regarding feasibility assessment (Table 2), a high number of the women indicated that SS was easy to use (87.9%), easy to insert and collect (79.5%), and user-friendly (91.6%). Especially the privacy of a self-sample compared to a CT specimen scored very high (92.8%). More than 80.0% of the women had confidence in the results of their self-taken sample. Furthermore, >85.0% of the women were willing to perform SS at the clinic or home, would go to a clinic that would provide the SS, and were even willing to pay for a NASS followed by an HPV test if it would be available over the counter.

**Table 2:**
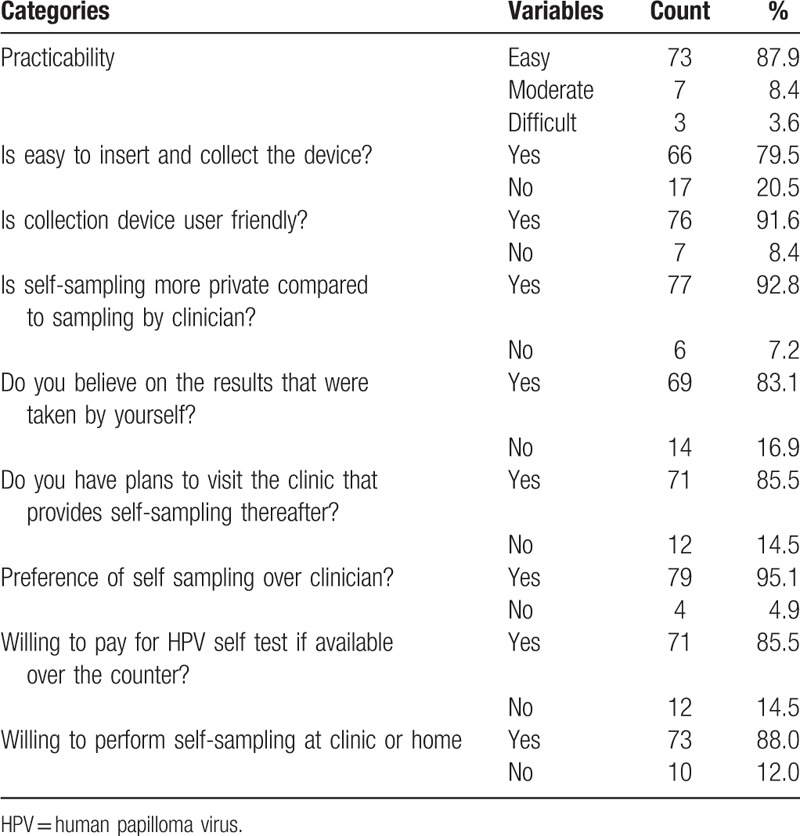
Acceptability and feasibility of vaginal self sampling by women enrolled in our cervical cancer screening study (N = 83).

### 3.3 Sample quality

Of 166 samples (2 specimens per women), 44 (26.6%) had not enough cells (>10 cells/μL) and were considered as samples with poor quality. According to Fisher exact test, there was no statistically significant difference in the number of samples with poor quality between the 2 sample groups (NASS: 19/83 and CT: 25/83) (*P* = .3794) (Table 3). For 17 women (20.5%), both the CT and the NASS samples were of poor quality (Table 4). These 17 women were excluded from further analysis.

**Table 3:**
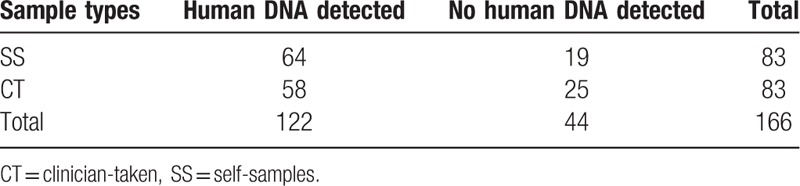
Sample quality comparsion between SS and CT samples (N = 166).

**Table 4:**
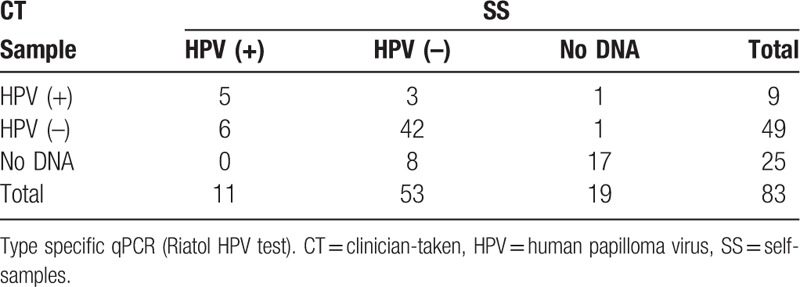
HPV test results of the SS and CT samples (N = 83).

### 3.4 HPV test results from the NASS and CT specimens

On all collected NASS and CT specimens, the RIATOL qPCR HPV genotyping test was performed. The HPV results of the remaining 66 women are presented in detail in Table 5. The overall prevalence of HPV was 22.7% (15/66). The prevalence of HR HPV was 18.2% (12/66) and LR HPV types 6.1% (4/66). The CT samples had an HPV prevalence of 15.5% (9/58) (all types), with a prevalence of 12.1% (7/58) for the high-risk types and 3.4% (2/58) for the low-risk types. The results from the NASS samples showed a somewhat higher prevalence of 17.2% (11/64), and 14.1% (9/64) and 4.7% (3/64) for all HPV types, HR, and LR types, respectively (Tables 4 and 5).

**Table 5:**
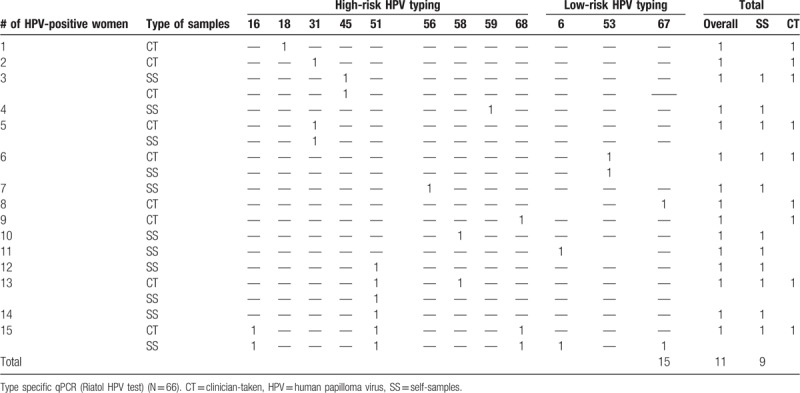
HPV distribution by type, of the SS and CT samples.

The overall agreement of HPV test results between NASS- and CT samples was moderate, with a kappa value of 0.58 (95% CI: 0.41–0.76). A total of 47/66 (71.2%) CT and NASS samples were in agreement in terms of HPV test results. From the 15 positive HPV samples, only 33.3% (5/15) were positive in both the NASS and CT sample, whereas for the HPV-negative results, there was 82.4% agreement (42/51).

The most prevalent HPV type was HR HPV51 (4/66, 6.1%), followed by HR HPV31, 58 and 68 and LR HPV6 and 67 which were all found twice (2/66, 3.0%). HPV16 was detected in 1 woman, in both the CT and the NASS sample (overall prevalence: 1/66 = 1.5%) and HPV18 in 1 woman, and only in the CT sample (overall/CT: 1/66 = 1.5%, NASS: 0%). Two women were coinfected with at least 2 HPV types (multiple infections: 2/66 = 3.0%). One woman of these 2 was coinfected with 5 HPV subtypes: HPV6, 16, 51, 67, and 68 (according to the NASS HPV DNA result). A total of 12 different HPV types were identified in this study, of the 18 HPV types that were tested for (Table 5).

### 3.5 Results from NASS and CT HPV test versus (VIA)

A total of 66 women underwent VIA. In 9 of 66 (13.6%) women, acetowhite lesions were visualized. When excluding the CT samples with poor sample quality, 5 of the 9 women with a positive VIA result were HPV-positive (sensitivity of 55.5% [95% CI: 26.6%–81.1%]) and 84.5% (49/58). However, 45 of the 49 women with no acetowhite lesions were also HPV-negative (specificity of 91.8% [95% CI: 80.8%–96.8%]). The overall agreement between HPV and VIA result from CT sample was 86.2% (Table 6).

**Table 6:**
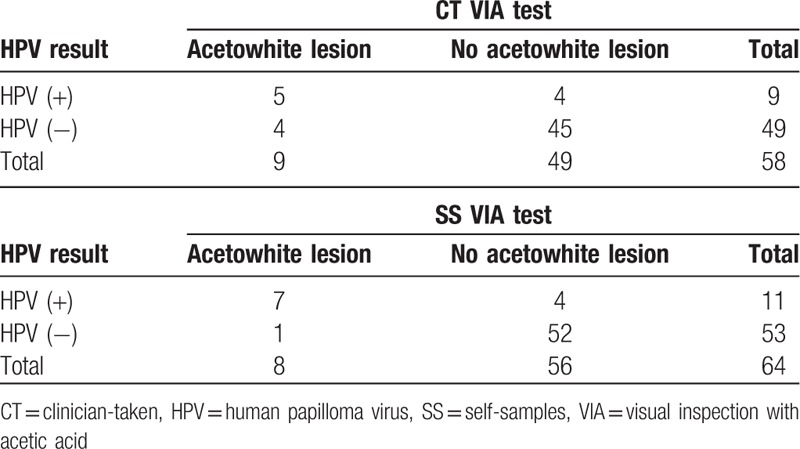
HPV test results versusVIA CT & SS HPV test results (N = 58, N = 64).

When excluding NASS samples with poor quality, 7 of the 8 women with acetowhite lesions were HPV-positive (sensitivity of 87.5% [95% CI: 52.9%–97.8%]) and 52 women of the 56 with no visual lesions were HPV-negative (sensitivity 92.8% [95% CI: 83.0%–97.2%]). The overall agreement between HPV and VIA result from NASS sample was 92.2% (Table 6).

### 3.6 Pearson *χ*^2^ test

Table 7 shows the result of Pearson *χ*^2^ test using the HR HPV test result (combined NASS and CT results) and all collected variables with HR HPV-negative status as the reference group. Having >2 lifetime sexual partners (*P* = .000447) and being VIA-positive were causally associated with an HR HPV-positive test result and not a difference by chance. Spontaneous abortion (*P* = .021) and being a housewife (*P* = .016) were also associated with HR HPV-positive results. Younger age groups (<40 years) showed a trend toward a correlation with a positive HPV test result (*P* = 0.058); there were about 19.6% (11/56) HR HPV-positive women in the age groups under 40 years, whereas only 10% (1/10) in the combined age groups above 40. Housewife and laborer were statistically associated with HR HPV (*χ*^2^ = 13.880 and *P* = .0016). No statistical association was found between HR HPV positivity and all other collected variables.

**Table 7:**
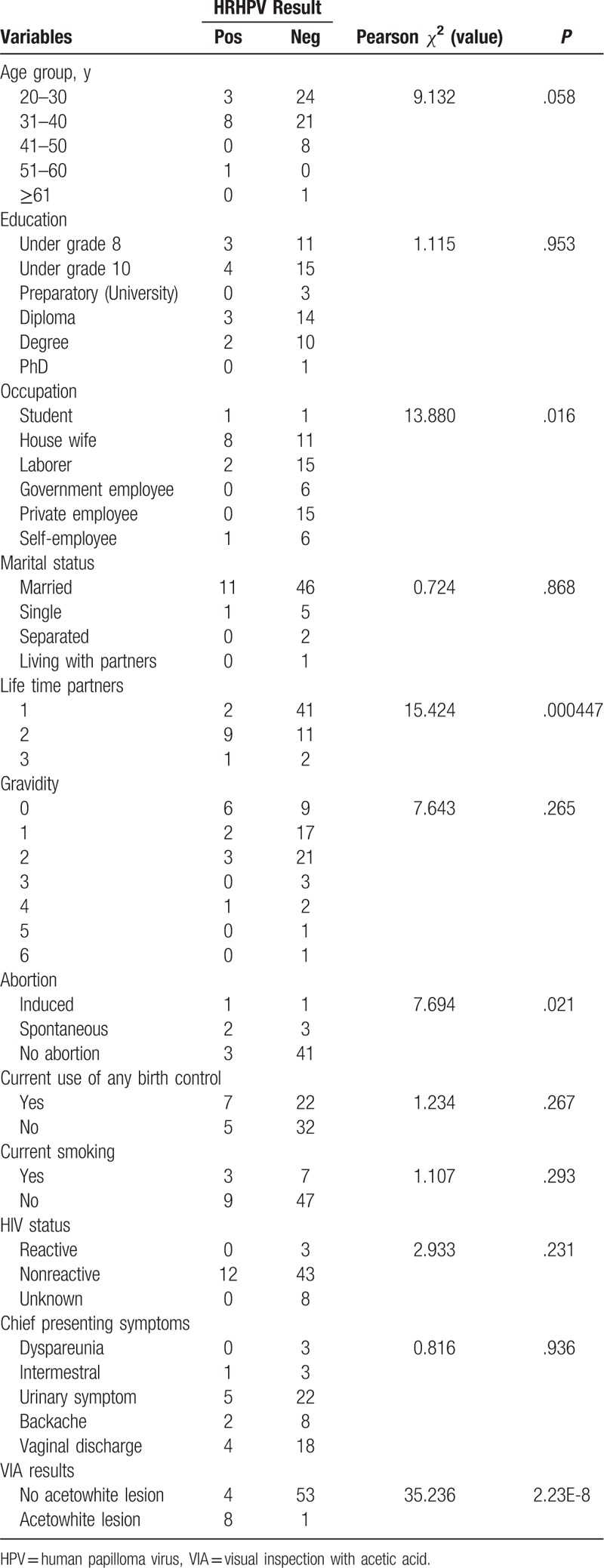
Pearson *χ*^2^ testof HR HPV test result (SS + CT results) with all studied variables (N = 66).

## 4 Discussion

### 4.1 Feasibility/acceptability of SS

Self-sampling devices are not commercially available in Ethiopia and not used for routine sample collection system on cervical cancer screening program. Thus, this study may be considered as first in kind to used SS collection device in Ethiopia. The acceptability of a SS device were very high and women felt SS device was easy to use, insert and collect, and user-friendly. Women were willing to perform the SS because of its private nature. Ghanaian women reported that 76.3% self-collected (SC) was very easy technique and easy to obtain; 57.7% preferred SS over CT sample and felt SC would increase their likelihood to access cervical cancer screening which was comparable percentage of women who felt same in our study too.^[10,11]^

Our study was further supported by data from Bolivia where SS was generally preferred over CT for a screening program based on HPV detection.^[12]^ Furthermore, a number of studies report that HPV SS was found to be highly acceptable and feasible among hard-to-reach women.

A study in El Salvador reported that SS revealed acceptability of 68%, although lower than reported in our study.^[13]^ Other studies from American-Indian and Hopi women have also supported our findings wherein samplings HPV testing was feasible and acceptable that may contribute to an increase of uptake.^[14]^

Most women showed a willingness to pay for SS services and believed their results which could be seen as a driving force for screening among hard-to-reach women.^[15]^

Almost the same percentage of women between our study and Japanese were reported; they would use SS again and found instructions easy to follow and reported no issues with the usability of the SS device. However, women in our study reported that they had confidence in the results of self-taken sampling unlike women who lacked confidence in the test.^[16]^

Similar studies supported our findings from Latinas and Haitian populations where women agreed HPV SS was faster, more private, easy to use, and would prefer to use it again.^[17]^ Furthermore, in German, SS considered being easy by 89.0% as well as user-friendly by 96.0% of the women.^[18]^ Therefore, Ethiopian women might use NASS service as an alternative option for fighting cervical cancer prevention.

### 4.2 HPV prevalence in general population

The authors reported that an overall HPV prevalence was 22.7% and a prevalence of HR HPV and LR HPV were 18.2% and 6.1%, respectively. HPV prevalence in Africa varied within a range of 12% to 46%.^[19]^ Two studies elsewhere in Ethiopia reported that the HR HPV prevalence was 17.3% and 15.8%.^[20,21]^ Thus, our study revealed HR HPV prevalence was consistent with sub-Saharan Africa report where ours is slightly higher. The overall HPV prevalence of SS and CT samples were 10.8% and 13.2% respectively. The authors could not find similar report on the prevalence of HR HPV among SS and doctor sampling which were 14.1% and 12.1%, respectively, in the general population of Ethiopia.

In Rwanda, the HR HPV prevalence was 19.0% which was slightly higher than our result.^[22]^ The prevalence of HR HPV in Dakar was 17.4% as compared to ours that is 18.2%, for which geographical areas and population difference could be a reason.^[23]^ The HR HPV prevalence in Cameroon was 18.5% that was comparable to our findings.^[24]^

A study from Northern Africa, a Muslim community, HPV infection was 6.3% (4.0% of them were HR types), with no significant variation by age.^[25]^ However, a study done by Traore et al^[26]^ in Burkina Faso revealed that HR HPV prevalence was 38.3%, which was twice of our result. Therefore, HPV prevalence was varied based on geographical areas and population segment as indicated in entire previous studies.

In a study done by Bruni et al,^[19]^ the estimated prevalence of HPV in Sub-Saharan Africa and global prevalence was 24.4% and 11.7%, respectively, which was almost comparable to our study.^[19]^ Further studies from 11 countries (Nigeria, India, Vietnam, Thailand, Korea, Colombia, Argentina, Chile, the Netherlands, Italy, and Spain) without cytological abnormalities were included and age-standardized HPV prevalence varied nearly 20 times between populations, from 1.4% in Spain to 25.6% in Nigeria where 22.5% HPV prevalence was presented in our study.^[27]^

### 4.3 HPV type distribution

From our study, the most prevalent HPV type was HPV51 and followed by HPV31, 58 and 68 (HR types) and HPV6 and 67 (LR types). Women were coinfected with at least 2 HPV types and the higher were coinfected with 5 HPV subtypes: HPV6, 16, 51, 67, and 68. A total of 12 HPV types were identified in this study of 19 HPV types tested. HPV16 was the most frequent genotype identified in samples from previous Ethiopia studies and HPV52, 58, and 18 were the second, third, and fourth common genotypes identified respectively, whereas in our study, HPV51 and 31 were the common genotypes identified.^[28]^ Thus, even within the same country, it possible to observe difference among segments of population.

In a study from South Africa, HPV16, 35, and 58 were the most common high-risk HPV types with no major differences in the type distribution by HIV status.^[29]^ In Mozambique, the most frequent were HPV51, HPV35, HPV18, HPV31, and HPV52. Also multiple infection were detected in normal cytology of types HPV51 and HPVs 16/18. And HPVs 51 and 35 were the two most common types.^[30]^

HPV-positive women in Europe were significantly more likely to be infected with HPV16 than those in sub-Saharan Africa. Heterogeneity between areas of Asia was significant were that supported by previous Ethiopian studies.^[27,31]^ In a study from Burkina Faso, HPV52, 33, and 59 were most identified genotypes, whereas HPV51, 31, and 58 were the most prevalent in our study.^[25]^

HPV52 (3.2%) was the most prevalent HPV type, followed by HPV31 (3.0%) and HPV16, 45, and 53 (all 2.8%).^[23]^ In a study from Nigeria, the prevalence of HPV35 and HPV16 was equally frequent.^[32]^ HPV16 was the most common type among the general population of Guinea (7.3%).^[33]^ Thus, these indicated that different genotypes were identified in different geographical areas and population.

### 4.4 HPV tests versus VIA

In this study, the overall agreement between SS HPV and VIA results was higher than CT results. The sensitivity between HPV and VIA test results was relatively higher on SS over CT samples. There was an almost equal specificity value found between SS and CT samples. A study from Cameroon indicated that the sensitivity and specificity of VIA/VILI among HPV-positive women were 80.0% and 44.0%, respectively, which were less compared to our study.^[24]^

A combination of HPV-based and VIA screen-and-treat approach may be feasible in a low-resource context and may contribute to improving the effectiveness of CC prevention programs. The combination of HPV-testing and VIA/VILI for CC screening might reduce over treatment.^[24]^

### 4.5 Agreement between NASS and CT HPV test

The overall agreement of HPV test results between NASS and CT samples was moderate, with a kappa value of 0.58. A study from Bolivia showed good agreement between self- and physician-collected samplesin where HR HPV detection (κ = 0.71) was higher as compared to our study.^[12]^ A study from Sub-Saharan Africa revealed that the overall HPV positivity agreement between self- and doctor was κ value of 0.52, respectively which had similar agreement with our study.^[34]^

## 5 Conclusions

There was a moderate agreement between NASS and CT sample for HPV detection. NASS might be used alternatively as sample collection strategy for HPV testing in cervical cancer screening program in Ethiopia; however, the quality sample should need improvement. NASS HPV testing is a valuable tool for the follow-up of women in low-resource settings. SC sampling may be used as alternative strategy to increase cervical cancer screening coverage in Ethiopia.

Although our study revealed that HPV51, 31, 16, 45, 52, and 58 were mostly identified, a large-scale study is required to study circulated genotypes in Ethiopia and select an appropriate HPV vaccine accordingly. Genotyping information could be important to guide vaccine policy. Our study was the first to report on HPV detection on SS using ThinPrep PreservCyt solution and may be used as a platform for similar studies in the future.

## 6 Limitation

Although this research was carefully prepared, we concluded that the sample size was small and not able to generalize.

## Acknowledgments

The authors acknowledged SAMH‘s staff for the support of sample collection activities and Hologic Inc Company, USA for donating ThinPrep PreservCyt solution, cytobrush, spatula through IPRH.

## Author contributions

**Conceptualization:** Eshetu Lemma Haile.

**Data curation:** Eshetu Lemma Haile.

**Formal analysis:** Eshetu Lemma Haile, Benoy Ina.

**Investigation:** Ransom Sharon.

**Methodology:** Eshetu Lemma Haile, Benoy Ina, Gurja Belay.

**Resources:** Van geertruyden Jean Pierre, Ransom Sharon.

**Supervision:** Eshetu Lemma Haile, Van geertruyden Jean Pierre, Bogers Johannes Paul.

**Writing – original draft:** Eshetu Lemma Haile.

**Writing – review & editing:** Eshetu Lemma Haile, Simoens Cindy, Benoy Ina, Van geertruyden Jean Pierre, Lebelo Ramokone Lisbeth, Bogers Johannes Paul.
